# An effective method for network module extraction from microarray data

**DOI:** 10.1186/1471-2105-13-S13-S4

**Published:** 2012-08-24

**Authors:** Priyakshi Mahanta, Hasin A Ahmed, Dhruba K Bhattacharyya, Jugal K Kalita

**Affiliations:** 1 Dept. of Comp. Sc. and Engg, Tezpur University, Napaam, Tezpur, India; 2 Dept. of Computer Science, University of Colorado, Colorado Springs, USA

## Abstract

**Background:**

The development of high-throughput Microarray technologies has provided various opportunities to systematically characterize diverse types of computational biological networks. Co-expression network have become popular in the analysis of microarray data, such as for detecting functional gene modules.

**Results:**

This paper presents a method to build a co-expression network (CEN) and to detect network modules from the built network. We use an effective gene expression similarity measure called NMRS (Normalized mean residue similarity) to construct the CEN. We have tested our method on five publicly available benchmark microarray datasets. The network modules extracted by our algorithm have been biologically validated in terms of Q value and p value.

**Conclusions:**

Our results show that the technique is capable of detecting biologically significant network modules from the co-expression network. Biologist can use this technique to find groups of genes with similar functionality based on their expression information.

## Introduction

The development of high-throughput Microarray technologies has provided a range of opportunities to systematically characterize diverse types of biological networks. Biological networks can be broadly classified as protein interaction networks [1-3], metabolic networks [4-6] and gene co-expression networks [7]. These networks provide an effective way to summarize gene and protein correlations. In this paper, we focus on gene co-expression networks, which is an undirected graph where nodes represent gene and nodes are connected by an edge if the corresponding gene pairs are significantly co-expressed. Gene co-expression networks provide the association between individual genes in terms of their expression similarity and a network-level view of the similarity among a set of genes. In co-expression networks, two genes are connected by an undirected edge if their activities have significant association, as computed using gene expression measurements such as Pearson correlation, Spearman correlation, mutual information. Compared to gene regulatory networks, a gene co-expression network is built upon gene neighborhood relations, which give interesting geometric interpretations of the network. One of the most important applications of gene co-expression networks is to identify functional gene modules [8] or network modules, which are represented by the strongly connected regions of the co-expression network.

### Problem formulation

Due to non-transitive nature of connections among genes, genes form a very complicated connectivity network with respect to a particular similarity measure in a gene expression data set. Such a connectivity network is often referred to as a co-expression network. A major use of this co-expression network is extraction of network modules that represent the strongly connected regions in the co-expression network. These modules may present highly co expressed genes, which are functionally similar.

In this paper, we propose an effective similarity measure for gene co-expression, develop an approach to prepare a co- expression network from a gene expression data set and mine the potential network modules from the built network. We aim to produce a graph, *G*={*V*,*E*} that presents the co-expression network with the following properties.

1. Each vertex *v*∈*V* represents a gene.

2. Each edge *e*∈*E* represents a connection between a pair of vertices *v*_1_,*v*_2_ where *v*_1_,*v*_2_ ∈*V.*

3. There is an edge between two vertices *v*_1_,*v*_2_ ∈*V* if the similarity of the genes corresponding to the vertices is more than a user defined threshold.

### Our contribution

We claim the following contributions in this paper.

• We introduce an effective gene similarity measure NMRS.

• We propose an approach to construct a co-expression network using NMRS.

• We develop a spanning tree based method to extract the potential network modules.

## Background

In the literature, a number of techniques have been proposed for gene co-expression network construction. When inferring co-expression networks from gene expression data, the algorithms take a gene expression dataset as primary input and then, by using a correlation-based proximity measure, constructs the corresponding co-expression networks. Frequently used correlation-based measures are Pearson correlation coefficient, Spearman correlation coefficient and Mutual information. Approaches such as [9,10] used Pearson correlation coefficient to extract the association among genes in a co-expression network. The Spearman correlation coefficient is used as a gene expression similarity measure to construct co-expression network in [10]. [11], Steuer et al. [12] reports the use of Mutual Information to find similarly expressed gene pairs in such networks. While some studies attempted to apply algorithms directly to the adjacency matrices of networks to partition network nodes into groups [13,14], other studies rely on special purpose algorithms for identifying subnetworks with certain properties [15].

Generally, in a co-expression network, the connections between genes are obtained from the absolute values of a co-expression measure. Several researchers have suggested to threshold this value of the co-expression measure to construct gene co-expression networks. There are two ways to pick a threshold: one way is picking a hard threshold (a number) based on the notion of statistical significance so that gene co-expression is encoded using binary information (connected=1, unconnected=0). The other way is called soft thresholding which weighs each connection by a number between 0 and 1. The drawbacks of hard thresholding include loss of information regarding the magnitude of gene connections and sensitivity to the choice of the threshold. Generally, hard thresholding results in unweighted networks while soft thresholding results in weighted networks.

## Methodology

To construct the gene co-expression network, we use the general framework proposed by [16]. A new effective gene similarity measure called NMRS is used to construct the distance matrix. We use a hard thresholding based signum function to construct the adjacency matrix from the distance matrix. A spanning tree based approach is used to detect network modules in the co-expression network. Extracted network modules are projected as functional categories of genes and these modules are validated using p value and Q value. Our approach is explained next.

### Define a gene expression measurement

To determine whether two genes have similar expression patterns, an appropriate similarity measure must be chosen [17]. To measure the level of concordance between gene expression profiles, we develop a gene co-expression measure called NMRS. The NMRS of gene *d*_1_=(*a*_1_, *a*_2_,…, *a_n_*) with respect to gene *d*_2_=(*b*_1_, *b*_2_,…, *b_n_*) is defined by 

where

#### NMRS as a metric

NMRS satisfies all the properties of a metric. We establish The non-negativity, symmetricity and triangular inequality properties for our measure in additional file 1.

#### Significance of NMRS

The most widely used proximity measures in gene expression data analysis are Euclidean distance, Pearson correlation coefficient, Spearman correlation coefficient, Mean squared residue etc. In co-expression network, the used proximity measure is expected to effectively detect the linear shifting patterns in the gene expression data. But none of the widely used proximity measures can satisfactorily serve this purpose. The Euclidean distance measures the distance between two data objects. But in this domain, the overall shapes of gene expression patterns (or profiles) are of greater interest than the individual magnitudes of each feature [18]. So Euclidean distance can not straight away detect shifting patterns, but bringing down all the genes to the same range of expression values can make this measure to detect shifting patterns. This normalization process involves an extra overhead. Along with shifting patterns Pearson correlation coefficient also detects scaling patterns and some other patterns which is normally not desired in a co-expression network and may lead to inclusion of genes which have considerable amount of difference between their expression levels. Spearman Rank Correlation Coefficient uses ranks to calculate correlation which can neither detect shifting patterns nor scaling patterns. Mean squared residue is good enough to detect shifting patterns, but the aggregate measure can not operate in a mutual mode, i.e. it can not find correlation between a pair of genes. A general comparison of these measures is presented in Table 1.

**Table 1:** Comparison of proximity measures

*Proximity measure*	*Mode*	*Normalization required*	*Detects shifting pattern*	*Detects scaling pattern*
Euclidian	Mutual	Yes	Yes	No
Pearson	Mutual	No	Yes	Yes
Spearman	Mutual	No	No	No
MSR	Aggregate	No	Yes	Yes
NMRS	Mutual	No	Yes	Yes

The table 1 presents the comparison of different proximity measure.

**Figure 1:**
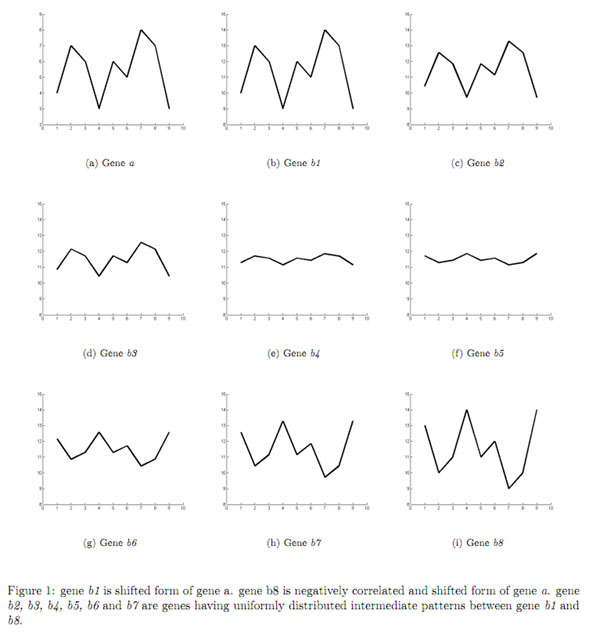
**Example patterns used for evaluation of proximity measures** The figure 1 presents the value of some example patterns that are used to demonstrate the superiority NMRS over other proximity measures viz. Euclidean distance, Pearson correlation coefficient and Spearman correlation coefficient.

**Figure 2:**
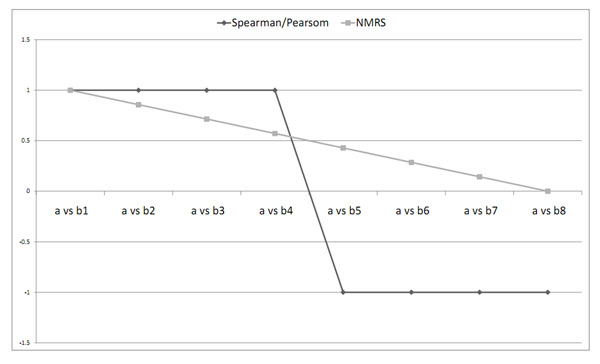
**NMRS and Pearson correlation coefficient among considered example patterns** The figure 2 presents NMRS and Pearson correlation coefficient of patterns *b1-b8* with that of *a.*

Let us consider a random gene pattern *a* as presented in Figure 1(a). Gene pattern *b1* in Figure 1(b) has a shifting relationship with gene *a.* Gene pattern *b8* in Figure 1(i) is a shifted as well as negatively correlated form of gene *a.* Figures 1(b)-1(h) present gene patterns *b2*, *b3*, *b4*, *b5*, *b6* and *b7* which are uniformly distributed intermediate patterns between genes *b1* and *b8.* Figure 2 shows Pearson, Spearman and NMRS correlation of gene patterns *b1-b8* with that of *a.* As usual the Spearman correlation was found to be concerned only about the rank information about the gene patterns. Interestingly, Pearson correlation was found to produce some undesired correlation values for the pairs *a and b2*, *a and b3*, *a and b4*, *a and b4*, *a and b5*, *a and b6* and *a and b7*, which are neither shifting nor scaling patterns. The values of these patterns are given in Table 2. Our measure is found to effectively distinguish patterns across this uniform distribution from a shifted pattern (with a value 1) to a shifted and negatively correlated pattern (with value 0) of a given pattern as can be seen in Figure 2.

**Table 2:** Gene pattern

*a*	4	7	6	3	6	5	8	7	3
*b1*	10	13	12	9	12	11	14	13	9

*b2*	10.4286	12.5714	11.8571	9.7143	11.8571	11.1429	13.2857	12.5714	9.7143

*b3*	10.8571	12.1429	11.7143	10.4286	11.7143	11.2857	12.5714	12.1429	10.4286

*b4*	11.2857	11.7143	11.5714	11.1429	11.5714	11.4286	11.8571	11.7143	11.1429

*b5*	11.7143	11.2857	11.4286	11.8571	11.4286	11.5714	11.1429	11.2857	11.8571

*b6*	12.1429	10.8571	11.2857	12.5714	11.2857	11.7143	10.4286	10.8571	12.5714

*b7*	12.5714	10.4286	11.1429	13.2857	11.1429	11.8571	9.7143	10.4286	13.2857

*b8*	13	10	11	14	11	12	9	10	14

The table 2 presents the random gene patterns for analysis of different proximity measures.

### Compute an adjacency matrix

An adjacency matrix is obtained using a signum function based hard thresholding approach which encodes edge information for each pair of nodes in the co-expression network. Two genes d*_i_* and d*_j_* are connected if Dist(d*_i_*,d*_j_*) >*δ*, a user defined threshold. Based on the connected pairs, an adjacency matrix is computed as

### Detect network modules

To detect subsets of nodes (modules) that are tightly connected to each other is an important aim of co-expression network analysis. In this paper, we use spanning trees and a topological overlap similarity measure [19] to find the network modules, since this measure is found to result in biologically meaningful modules. A tree *T* is a spanning tree of a connected graph *G* if *T* is a subgraph of *G* and it contains all vertices of *G.* We use Prim’s algorithm [20] to find a spanning tree of a undirected graph. However, unlike traditional Prim’s algorithm we find a spanning tree with maximum weight. For unweighed networks (i.e. a*_ij_* = 1 or = 0), the topological overlap matrix is defined by(1)

where *l_ij_* = ∑*_u_a_iu_a_ij_*, and *k_i_* = ∑*_u_a_iu_* is the node connectivity.

### Extract useful information

Extraction of useful biological information is one of the main usages of gene co-expression networks. From the constructed network, one can explore various important information such as functionality and pathways of genes, essential genes susceptible to diseases.

## Proposed algorithm: Module Miner

Module Miner takes NMRS threshold, *δ*, as a input and works on a microarray gene data and constructs the gene co-expression network and finally network modules are extracted from the network. Our approach uses an effective similarity measure NMRS to form a co-expression network using signum function. The co-expression network is further explored to mine the potential network modules using a spanning tree based method and a connectivity measure called *Topological Overlap Matrix.*

The symbols provided in Table 3 and definitions given below are useful in discussing the proposed Module Miner algorithm.

**Table 3:** Symbolic representation

*SYMBOL*	*MEANING*
*D*	The gene expression matrix
*d_i_*	i*^th^*gene in *D*
*δ*	Signum threshold
*G*	Co-expression network
*V*	Set of vertices in G
*E*	Set of edges in G
*Dist*	Distance matrix
*Dist*(*d_i_*, *d_j_*)	NMRS distance between genes d*_i_*, d*_j_*∈D
*Adj*	Adjacency matrix
*Adj*(*v_i_*,*v_j_*)	1 if v*_i_* and v*_j_* are connected by an edge 0 otherwise
*G^con^*	Set of connected region
	i*^th^* connected region
	Set of vertices in i*^th^* connected region
	Set of edges in i*^th^* connected region
	Adjacency matrix of the i*^th^* connected region
	i*^th^* network module
*D^net^*	Set of network modules obtained from *G*
*TOM*(*v_i_*,*v_j_*)	Topological Matrix value between vertices v*_i_* and v*_j_*
*TOM*(*V*_1_)	Average TOM of the set of vertices V_1_
	TOM for i*^th^* connected region
	Maximum spanning tree obtained from i*^th^* connected region
	Set of edges in

The table 3 describes the various symbols that is used in *ModuleMiner.*

**Definition 1 ***A ****CEN ****can be defined by an undirected*, *graph G*={*V*,*E*} *where each v*∈*V corresponds to a gene and each edge e*∈*E corresponds a pair of genes d_i_*, *d_j_*∈*D such that Dist*(*d*_i_, *d_j_*)≥*δ.*

**Definition 2 *Connected regions ****in a CEN are parts of the network where each pair of vertices is connected by a path. The i^th^ connected region extracted from G can be defined as a graph **where **and **such that for any vertex *, *there is at least one vertex **which are connected by an edge **.*

**Definition 3 *Maximum spanning tree****of a weighted graph is a spanning tree obtained from i^th^ connected region*, *can be defined as *, *where the sum of TOM values associated with edges in **is maximum compared to other spanning trees.*

**Definition 4 *Network modules ****are highly connected regions of the co-expression network. The i^th^ network module derived from j^th^ connected region **is defined as a set of vertices **if*

• *and **where **are obtained by removing the weakest edge of the maximum spanning tree built for the subgraph of G consisting of vertex set **or*

• *TOM*(*V*_3_)>*TOM*(*V*_4_) *and **where*, *are obtained by removing the weakest edge of the maximum spanning tree built for the subgraph of G consisting of vertex set V*_4_.

### Algorithm: *Module Miner*

The pseudo code of Module Miner is presented in Algorithm 1. In the pseudo code, lines 1-4 extracts the connected regions from the gene expression data. Lines 5-25 process each of the connected regions to extract the network modules. A maximum spanning tree is constructed using Prim’s algorithm [20] from a connected region with weights defined by topological overlap matrix in lines 6-8. Lines 9-10 find and remove the weakest edge from the spanning tree. Removal of this edge from the spanning tree leads to two subtrees which are processed in lines 11-23 to form either a connected module or a new connected region.

### Algorithm complexity

The complexity of different steps of our method is presented in this section.

• The preparation of the distance matrix involves a complexity of O(*n*×*n-1*)/2, where *n* is the number of genes.

• Finding connected regions from the co-expression network requires a complexity of O(*n*).

• Computation of the TOM matrix involves a complexity of O(*n_c_*×(d*_c_*×(d*_c_*-1)/2)), where n*_c_* is the total number of connected regions and d*_c_* is the average number of genes in the connected regions.

• Finding a maximum spanning tree consumes a complexity of .

## Experimental results

We implemented the Module Miner algorithm in MATLAB and tested it on five benchmark microarray datasets mentioned in Table 4. The test platform was a SUN workstation with Intel(R) Xenon(R) 3.33 GHz processor and 6 GB memory running Windows XP operating system.

**Table 4:** Datasets used for evaluating *ModuleMiner*

Serial. No	Dataset	No. of Genes/ No. of Conditions	Source
1	Yeast Sporulation	474/17	http://cmgm.stanford.edu/pbrown/sporulation/index.html
2	Yeast Diauxic Shift	689/72	Sample gene in expander
3	Subset of Yeast Cell Cycle	384/17	http://faculty.washington.edu/kayee/cluster
4	Arabidopsis Thaliana	138/8	http://homes.esat.kuleuven.be/~sistawww/bioi/thijs/Work/Clustering.html
5	Rat CNS	112/9	http://faculty.washington.edu/kayee/cluster

The table 4 gives the description of various datasets used in *ModuleMiner.*

### Validation

The performance of Module Miner on the five publicly available benchmark microarray dataset is measured in terms of p value and Q value.

#### p value

Biological significance of the sets of genes included in the extracted network modules are evaluated based on p values [21]. p value signifies how well these genes match with different Gene Ontology(GO) categories. A cumulative hypergeometric distribution is used to compute the p value. A low p-value of the set of genes in a network module indicates that the genes belong to enriched functional categories and are biologically significant. From a given GO category, the probability p of getting k or more genes within a cluster of size n, is defined as(2)

where f and g denote the total number of genes within a category and within the genome respectively.

To compute p-value, we used a tool called FuncAssociate [22]. FuncAssociate computes the hyper geometric functional enrichment score based on Molecular Function and Biological Process annotations. The enriched functional categories for some of the network modules obtained by Module miner on the datasets are presented in Tables 5 and 6. The co-expression network modules produced by Module Miner contains the highly enriched cellular components of *DNA replication*, *DNA repair*, *DNA metabolic process*, *response to DNA damage stimulus*, *nuclear nucleosome*, *nucleosome*, *nucleosome assembly*, *protein-DNA complex*, *cell wall assembly*, *meiosis*, *cell differentiation*, *sporulation resulting in formation of a cellular spore*, *sporulation*, *anatomical structure formation involved in morphogenesis*, *cellular developmental process*, *reproductive cellular process*, *cell cycle phase*, *developmental process*, *cell cycle process*etc with p-values of **7.69 × 10^–27^, 3.93 × 10^–25^, 1.03 × 10^–26^, 1.23 × 10^–23^, 2.32 × 10^–28^, 5 .12 × 10^–27^, 7.27 × 10^–23^, 2.06 × 10^–20^, 3.84 × 10^–32^, 1.41 × 10^–31^, 1.19 × 10^–38^, 9.65 × 10^–36^, 1.34 × 10^–20^, 2.52 × 19^–34^, 1.93 × 10^–28^ and 6.91 × 10^–27^** being the highly enriched one. From the given p values, we can conclude that Module Miner shows a good enrichment of functional categories and therefore project a good biological significance.

**Table 5:** P-value of one of the network modules of Dataset 2

*P-value*	*GO number*	*GO category*
2.32E-28	GO:0000788	nuclear nucleosome
5.12E-27	GO:0000786	nucleosome
7.27E-23	GO:0006334	nucleosome assembly
2.06E-20	GO:0032993	protein-DNA complex
8.61E-19	GO:0034728	nucleosome organization
1.14E-18	GO:0065004	protein-DNA complex assembly
1.12E-17	GO:0006333	chromatin assembly or disassembly
4.12E-16	GO:0005694	chromosome
2.49E-14	GO:0044454	nuclear chromosome part
1.70E-13	GO:0031298	replication fork protection complex
9.47E-14	GO:0006325	chromatin organization
6.78E-13	GO:0044427	chromosomal part
2.32E-12	GO:0034622	cellular macromolecular complex assembly

The table 5 gives the p value of one of the network modules of Dataset 2.

**Table 6:** p-value of one of the network modules of Dataset 3

*P-value*	*GO number*	*GO category*
3.93E-25	GO:0006281	DNA repair
1.03E-26	GO:0006259	DNA metabolic process
1.23E-23	GO:0006974	response to DNA damage stimulus
7.69E-27	GO:0006260	DNA replication
6.94E-19	GO:0007049	cell cycle
5.55E-16	GO:0005634	nucleus
8.53E-18	GO:0044454	nuclear chromosome part
1.51E-17	GO:0022402	cell cycle process
3.53E-17	GO:0000079	regulation of cyclin-dependent protein kinase activity
5.72E-15	GO:0045859	regulation of protein kinase activity
5.16E-16	GO:0005657	replication fork

The table 6 gives the p value of one of the network modules of Dataset 3.

#### Q value

The Q-value [23] for a particular gene G is the proportion of false positives among all genes that are as or more extremely differentially expressed. Equivalently, the Q-value is the minimal False Discovery Rate(FDR) at which this gene appears significant. The GO categories and Q-values from a FDR corrected hypergeometric test for enrichment are reported in GeneMANIA. Q-values are estimated using the Benjamini Hochberg procedure. Different GO categories of the co-expression networks produced by Module miner are displayed up to a Q-value cutoff of 0.1 in Table 7, 8, 9, 10 and 11. The co-expression network modules produced by Module Miner contains the highly enriched cellular components of *sporulation resulting in formation of a cellular spore*, *spore wall assembly*, *ascospore wall assembly*, *ascospore formation*, *sexual sporulation*, *spore wall biogenesis*, *ascospore wall biogenesis*, *sexual sporulation resulting in formation of a cellular spore*, *cell development cell wall assembly*, *reproductive process in single-celled organism*, *cell differentiation*, *fungal-type cell wall biogenesis*, *reproductive developmental process*, *reproductive process*, *reproductive cellular process*, *reproduction of a single-celled organism*, *cell wall biogenesis*, *sexual reproduction*, *anatomical structure development*, *anatomical structure morphogenesis* , *M phase*, *meiotic cell cycle*, *meiosis*, *M phase of meiotic cell cycle*etc with Q-values of **1.53 × 10^–34^, 3.43 × 10^–33^, 2.59 × 10^–32^, 6.93 × 10^–30^, 1.40 × 10^–29^, 1.86 × 10^–25^, 9.90 × 10^–25^, 1.25 × 10^–24^, 4.83 × 10^–24^, 5.45 × 10^–24^, 2.10 × 10^–23^, 1.62 × 10^–21^, 2.74 × 10^–21^** being the highly enriched one. From the results of Q value, we arrive at the conclusion that the genes in a network module cluster obtained by Module Miner seem to be involved in similar functions.

**Table 7:** Q-value of one of the network modules of Dataset 3

*GO annotation*	*Q value*
DNA replication	1.93E-21
DNA repair	1.93E-21
response to DNA damage stimulus	2.17E-20
DNA-dependent DNA replication	3.07E-19
replication fork	6.27E-19
nuclear chromosome	1.23E-17
mitotic sister chromatid cohesion	5.51E-17
nuclear replication fork	9.37E-17
nuclear chromosome part	2.00E-16
sister chromatid cohesion	5.13E-15

The table 7 gives the Q value of one of the network modules of Dataset 3.

**Table 8:** Q-value of one of the network modules of Dataset 1

*GO annotation*	*Q value*
cytosolic ribosome	1.43E-52
cytosolic part	3.26E-48
structural constituent of ribosome	2.11E-44
ribosomal subunit	1.16E-42
cytosolic large ribosomal subunit	2.65E-36
large ribosomal subunit	1.47E-27
preribosome	2.96E-23
cytosolic small ribosomal subunit	3.71E-17
90S preribosome	8.48E-16

The table 8 gives the Q value of one of the network modules of Dataset 1.

**Table 9:** Q-value of one of the network modules of Dataset 1

*GO annotation*	*Q value*
sporulation resulting in formation of a cellular spore	1.53E-34
sporulation	1.53E-34
anatomical structure formation involved in morphogenesis	1.53E-34
spore wall assembly	3.43E-33
ascospore wall assembly	3.43E-33
ascospore formation	3.43E-33
sexual sporulation	3.43E-33
spore wall biogenesis	3.43E-33
ascospore wall biogenesis	3.43E-33
sexual sporulation resulting in formation of a cellular spore	3.43E-33
cell development	3.43E-33
cell wall assembly	8.88E-33
reproductive process in single-celled organism	2.59E-32
cell differentiation	8.40E-32
fungal-type cell wall biogenesis	6.93E-30
reproductive developmental process	1.40E-29
reproductive process	1.86E-25
reproductive cellular process	1.86E-25
reproduction of a single-celled organism	9.90E-25
cell wall biogenesis	1.25E-24
sexual reproduction	4.83E-24
anatomical structure development	5.45E-24
anatomical structure morphogenesis	5.45E-24
M phase	2.10E-23
meiotic cell cycle	1.62E-21
meiosis	2.74E-21
M phase of meiotic cell cycle	2.74E-21

The table 9 gives the Q value of one of the network modules of Dataset 1.

**Table 10:** Q-value of one of the network modules of Dataset 4

*GO annotation*	*Q value*
synaptic transmission	1.29E-13
glutamate receptor activity	3.77E-11
synapse	6.68E-08
regulation of synaptic transmission	3.06E-07
regulation of transmission of nerve impulse	4.00E-07
regulation of neurological system process	7.07E-07
regulation of system process	5.38E-05
synapse part	8.11E-04
cell projection part	9.46E-04

The table 10 gives the Q value of one of the network modules of Dataset 4.

**Table 11:** Q-value of one of the network modules of Dataset 5

*GO annotation*	*Q value*
regulation of synaptic transmission	6.438756E-7
regulation of transmission of nerve impulse	9.297736E-7
regulation of neurological system process	1.533111E-6
intermediate filament cytoskeleton organization	2.056912E-6
intermediate filament-based process	5.218967E-6
neurofilament cytoskeleton	1.109702E-5
intermediate filament organization	1.454524E-5
synapse part	2.543099E-5
growth factor binding	2.571707E-5
intermediate filament	2.938762E-5
positive regulation of neurogenesis	9.6019E-5

The table 11 gives the Q value of one of the network modules of Dataset 5.

**Figure 3:**
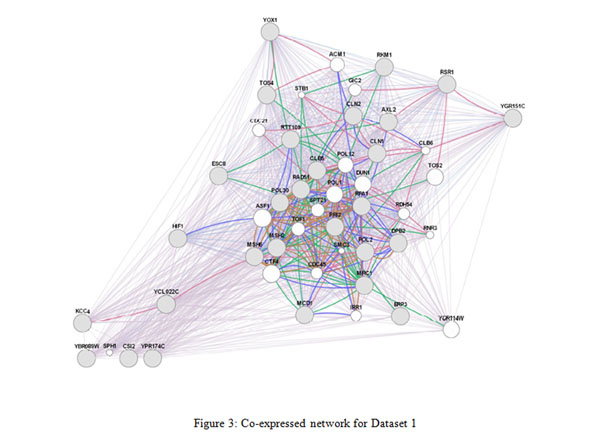
**Visualization of co-expressed network** The figure3 presents co-expressed network by GeneMANIA for Dataset1.

**Figure 4:**
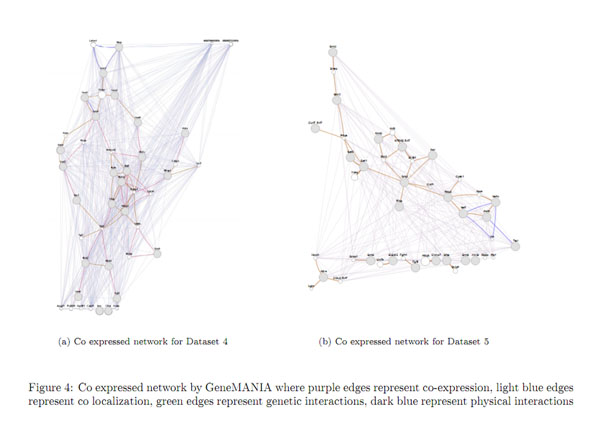
**Visualization of co-expressed network** The figure 4 presents co-expressed network by GeneMANIA for Dataset2 and Dataset3.

**Figure 5:**
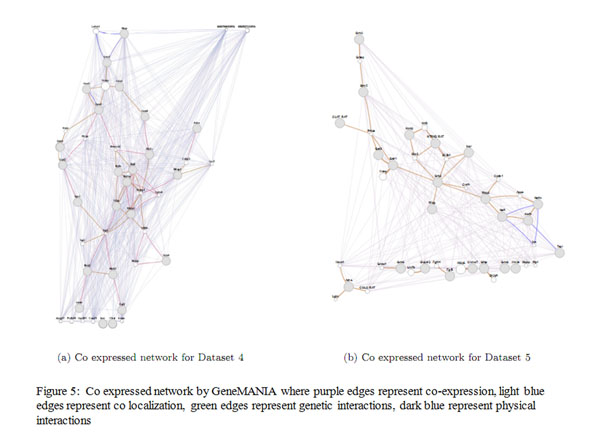
**Visualization of co-expressed network** The figure 5 presents co-expressed network by GeneMANIA for Dataset4 and Dataset5.

We have used GeneMANIA [24] which is a flexible, user-friendly web interface for generating hypotheses about gene function, analyzing gene lists and prioritizing genes for functional assays. Given a query list, GeneMANIA extends the list with functionally similar genes that it identifies using available genomics and proteomics data. GeneMANIA displays results as an interactive network, illustrating the functional relatedness of the query and retrieved genes. GeneMANIA currently supports different networks including co-expression, physical interaction, genetic interaction, co-localization etc. On a given set of genes and their connectivity information, GeneMANIA also assigns coverage ratios as percentage to each of these networks with respect to the annotated genes in the genome. The percentage of co-expression on network modules produced by Module Miner is given in Table 12. The values are obtained by choosing the default network weighting option i.e. **automatically selected weighing method**. Visualization of some of the co-expression networks generated by GeneMANIA for the datasets are presented in Figures 3, 4, 5.

**Table 12:** The weightage of co-expression by Module Miner

*Datasets*	*Network Modules*	*Percentage*
Dataset1	C1	99.57%
	C2	88.89%

Dataset2	C1	59.23%
	C2	77.27%

Dataset3	C1	92.13%
	C2	88.89%
	C3	92.33%
	C4	67.65%

Dataset4	C1	81.85%

Dataset5	C1	76.62%

The table 12 gives the percentage of co-expression on network modules produced by Module Miner.

## Conclusion and future work

In this paper, an effective gene expression similarity measure NMRS is introduced, which is used to construct the co-expression network through a signum function based hard thresholding scheme. Finally, network modules are extracted from the network using maximum spanning tree and topological overlap matrix. However, soft thresholding method can be used to construct the adjacency matrix to reduce information loss. Generalized Topological Overlap Measure [25] can be used instead of Topological Overlap Measure to get more accurate results. There is scope to design supervised models to derive gene regulatory network from the co-expression network.

## Competing interests

The author(s) declare that they have no competing interests.

## Supplementary Material

Additional file 1 **NMRS as a metric** This additional file 1 presents the proofs of different metric properties of NMRS measure. Click here for file
