# Supplementary material for: An effective method for network module extraction from microarray data
Source: BMC Bioinformatics. 2012 Aug 24;13(Suppl 13):S4. doi: 10.1186/1471-2105-13-S13-S4 (PMC3426802; doi:10.1186/1471-2105-13-S13-S4)
Supplement: Additional file 1 — NMRS as a metric This additional file 1 presents the proofs of different metric properties of NMRS measure. [file 1471-2105-13-S13-S4-S1.pdf]

Metric properties of NMRS

(a) **Non-negativity**
To satisfy non-negativity property, NMRS of two genes should be always greater than zero,
i.e., NMRS(a,b)>0, where a and b are two gene profiles.
Proof:

$$NMRS(a,b) = 1 - \frac{\sum_{i=1}^{p} |a_i - a_{mean} - b_i + b_{mean}|}{2 \times max\{\sum_{i=1}^{p} |(a_i - a_{mean})|, \sum_{i=1}^{p} |(b_i - b_{mean})|\}}$$

Suppose

$$\begin{aligned} \frac{\sum_{i=1}^{p} |a_i - a_{mean} - b_i + b_{mean}|}{2 \times max\{\sum_{i=1}^{p} |(a_i - a_{mean})|, \sum_{i=1}^{p} |(b_i - b_{mean})|\}} &< 0. \text{ This implies} \\ \sum_{i=1}^{p} |a_i - a_{mean} - b_i + b_{mean}| &< 0. \end{aligned}$$

Since absolute value of a quantity is always positive, this contradicts the supposition. Hence, the supposition is false. So

$$\frac{\sum_{i=1}^{p} |a_i - a_{mean} - b_i + b_{mean}|}{2 \times max\{\sum_{i=1}^{p} |(a_i - a_{mean})|, \sum_{i=1}^{p} |(b_i - b_{mean})|\}} > 0. \quad (1)$$

Again, suppose

$$\frac{\sum_{i=1}^{p} |a_i - a_{mean} - b_i + b_{mean}|}{2 \times max\{\sum_{i=1}^{p} |(a_i - a_{mean})|, \sum_{i=1}^{p} |(b_i - b_{mean})|\}} > 1$$

$$\begin{aligned} \implies & \sum_{i=1}^{p} |a_i - a_{mean} - b_i + b_{mean}| > 2 \times max\{\sum_{i=1}^{p} |(a_i - a_{mean})|, \sum_{i=1}^{p} |(b_i - b_{mean})|\} \\ \implies & \sum_{i=1}^{p} |(a_i - a_{mean}) - (b_i - b_{mean})| > 2 \times max\{\sum_{i=1}^{p} |(a_i - a_{mean})|, \sum_{i=1}^{p} |(b_i - b_{mean})|\} \quad (2) \end{aligned}$$

According to Modulo property $|a - b| \geq |a| - |b|$,

$$\implies \sum_{i=1}^{p} |(a_i - a_{mean}) - (b_i - b_{mean})| > \sum_{i=1}^{p} |(a_i - a_{mean})| - \sum_{i=1}^{p} |(b_i - b_{mean})|.$$

From equation (2)

$$\begin{aligned} \sum_{i=1}^{p} |(a_i - a_{mean}) - (b_i - b_{mean})| &> \sum_{i=1}^{p} |(a_i - a_{mean})| - \sum_{i=1}^{p} |(b_i - b_{mean})| \\ &> 2 \times max\{\sum_{i=1}^{p} |(a_i - a_{mean})|, \sum_{i=1}^{p} |(b_i - b_{mean})|\}. \end{aligned}$$

Since difference between two positive absolute quantities can not be greater than the two times of maximum absolute value of either, this contradicts the supposition. Hence, the supposition is false. So,

$$\frac{\sum_{i=1}^{p} |a_i - a_{mean} - b_i + b_{mean}|}{2 \times max\{\sum_{i=1}^{p} |(a_i - a_{mean})|, \sum_{i=1}^{p} |(b_i - b_{mean})|\}} \leq 1. \quad (3)$$

From equation (1) and equation (6)

$$0 < \frac{\sum_{i=1}^{p} |a_i - a_{mean} - b_i + b_{mean}|}{2 \times max\{\sum_{i=1}^{p} |(a_i - a_{mean})|, \sum_{i=1}^{p} |(b_i - b_{mean})|\}} < 1.$$

Multiplying by -1,

$$0 > -\frac{\sum_{i=1}^{p} |a_i - a_{mean} - b_i + b_{mean}|}{2 \times max\{\sum_{i=1}^{p} |(a_i - a_{mean})|, \sum_{i=1}^{p} |(b_i - b_{mean})|\}} \geq -1.$$

Adding 1 in both sides, (4)

$$\implies 1 + 0 > 1 - \frac{\sum_{i=1}^{p} |a_i - a_{mean} - b_i + b_{mean}|}{2 \times max\{\sum_{i=1}^{p} |(a_i - a_{mean})|, \sum_{i=1}^{p} |(b_i - b_{mean})|\}} \geq 1 - 1$$

$$\implies 1 > 1 - \frac{\sum_{i=1}^{p} |a_i - a_{mean} - b_i + b_{mean}|}{2 \times max\{\sum_{i=1}^{p} |(a_i - a_{mean})|, \sum_{i=1}^{p} |(b_i - b_{mean})|\}} \geq 0$$

$$\implies 1 > NMRS(a, b) \geq 0. \quad (5)$$

Hence, we have proved that NMRS satisfies the non-negativity property. Moreover, for any two genes, the value of NMRS always lies between 0 and 1.

(b) **Symmetricity**
To satisfy the symmetricity property, for any two genes a and b, NMRS(a,b) should be equal to NMRS(b,a), i.e., NMRS(a,b)=NMRS(b,a).
Proof:

$$NMRS(a, b) = 1 - \frac{\sum_{i=1}^{p} |a_i - a_{mean} - b_i + b_{mean}|}{2 \times max\{\sum_{i=1}^{p} |(a_i - a_{mean})|, \sum_{i=1}^{p} |(b_i - b_{mean})|\}} \quad (6)$$

$$NMRS(b, a) = 1 - \frac{\sum_{i=1}^{p} |b_i - b_{mean} - a_i + a_{mean}|}{2 \times max\{\sum_{i=1}^{p} |(a_i - a_{mean})|, \sum_{i=1}^{p} |(b_i - b_{mean})|\}} \quad (7)$$

According to modulo property,

$$|a - b| = | - (b - a)| = |b - a|$$

$$\implies \sum_{i=1}^{p} |a_i - a_{mean} - b_i + b_{mean}| = \sum_{i=1}^{p} (-| - a_i + a_{mean} + b_i - b_{mean}|)$$

$$\implies \sum_{i=1}^{p} |a_i - a_{mean} - b_i + b_{mean}| = \sum_{i=1}^{p} (| - a_i + a_{mean} + b_i - b_{mean}|)$$

$$\implies \sum_{i=1}^{p} |a_i - a_{mean} - b_i + b_{mean}| = \sum_{i=1}^{p} (|b_i - b_{mean} - a_i + a_{mean}|)$$

$$\implies 1 - \sum_{i=1}^{p} |a_i - a_{mean} - b_i + b_{mean}| = 1 - \sum_{i=1}^{p} (|b_i - b_{mean} - a_i + a_{mean}|)$$

$$\implies NMRS(a, b) = NMRS(b, a) \text{(Denominator of both equation (6) and (7) are same)} \quad (8)$$

Hence, we have proved that NMRS satisfies the symmetricity property.

(c) **Subadditivity or Triangle Inequality**
To satisfy triangular inequality property, for any three genes a, b and c, the following condition should hold:
NMRS(a,b)+NMRS(b,c)≥NMRS(a,c).

Proof:
From equation (5), we have

$$0 \leq NMRS(a,b) < 1 \tag{9}$$

$$0 \leq NMRS(b,c) < 1 \tag{10}$$

$$0 \leq NMRS(a,c) < 1 \tag{11}$$

From (9)+(10), we have

$$0 \leq NMRS(a,b + NMRS(b,c) < 1 + 1$$

$$0 \leq NMRS(a,b) + NMRS(b,c) < 2 \tag{12}$$

From (11) and (12),

$$NMRS(a,b) + NMRS(b,c) \geq NMRS(a,c)$$

Hence it is proved that NMRS satisfies the triangle inequality property.
